# Behavioural effects of APH199, a selective dopamine D4 receptor agonist, in animal models

**DOI:** 10.1007/s00213-023-06347-1

**Published:** 2023-02-28

**Authors:** Daria Chestnykh, Fabian Graßl, Canice Pfeifer, Jonas Dülk, Chiara Ebner, Mona Walters, Stephan von Hörsten, Johannes Kornhuber, Liubov S. Kalinichenko, Markus Heinrich, Christian P. Müller

**Affiliations:** 1 Department of Psychiatry and Psychotherapy, University Clinic, Friedrich-Alexander-University of Erlangen-Nuremberg, Schwabachanlage 6, 91054 Erlangen, Germany; 2 Department of Chemistry and Pharmacy, Friedrich-Alexander-University of Erlangen-Nuremberg, Nikolaus-Fiebiger-Str. 10, 91058 Erlangen, Germany; 3 Department of Experimental Therapy, Preclinical Experimental Center, Friedrich-Alexander-University of Erlangen-Nuremberg, Palmsanlage 5, 91054 Erlangen, Germany; 4 Centre for Drug Research, University Sains Malaysia, Penang, Minden Malaysia

**Keywords:** DRD4 agonist, APH199, Anxiety, Depression, Schizophrenia, Psychosis, Alcohol

## Abstract

**Rationale:**

The dopamine D4 receptors (DRD4) play a key role in numerous brain functions and are involved in the pathogenesis of various psychiatric disorders. DRD4 ligands have been shown to moderate anxiety, reward and depression-like behaviours, and cognitive impairments. Despite a series of promising but ambiguous findings, the therapeutic advantages of DRD4 stimulation remain elusive.

**Objectives:**

The investigation focused on the behavioural effects of the recently developed DRD4 agonist, APH199, to evaluate its impact on anxiety, anhedonia, behavioural despair, establishment and retrieval of alcohol reinforcement, and amphetamine (AMPH)-induced symptoms.

**Methods:**

Male C57BL/6 J mice and Sprague–Dawley rats were examined in five independent experiments. We assessed APH199 (0.1–5 mg/kg, i.p.) effects on a broad range of behavioural parameters in the open field (OF) test, conditioned place preference test (CPP), elevated plus maze (EPM), light–dark box (LDB), novelty suppressed feeding (NSF), forced swim test (FST), sucrose preference test (SPT), AMPH-induced hyperlocomotion test (AIH), and prepulse inhibition (PPI) of the acoustic startle response in AMPH-sensitized rats.

**Results:**

APH199 caused mild and sporadic anxiolytic and antidepressant effects in EPM and FST, but no remarkable impact on behaviour in other tests in mice. However, we found a significant increase in AMPH-induced hyperactivity, suggesting an exaggeration of the psychotic-like responses in the AMPH-sensitized rats.

**Conclusions:**

Our data challenged the hypothesis of the therapeutic benefits of DRD4 agonists, pointing out a possible aggravation of psychosis. We suggest a need for further preclinical studies to ensure the safety of antipsychotics with DRD4 stimulating properties.

## Introduction

Dopamine receptors belonging to the D2-like receptor family are involved in a great variety of essential brain functions, including primary motivation, locomotion, and cognition (Missale et al. 1998; Beaulieu and Gainetdinov 2011; Fernandes et al. 2012). The members of the D2-like family are inhibitory seven transmembrane G-protein coupled receptors and are divided into three subtypes: D2, D3, and D4 dopamine receptors. The latest was described and cloned only in 1991 (Van Tol et al. 1991) and remains the least well-studied.

The focus on dopamine receptors D4 (DRD4) had been magnified lately when a remarkable human *DRD4* gene polymorphism was discovered. The polymorphic gene variants, or Variable Number Tandem Repeats (VNTR), emerge from a different number of 48 base pairs in the third exon. The highest prevalence was indicated for 4 (D4.4), followed by 7 (D4.7) and 2 times (D4.2) of the repeats (Chang et al. 1996; Lichter et al. 1993; Chestnykh et al. 2021). Specific VNTRs have been associated with the occurrence of psychiatric disorders, sensitivity to pharmacotherapy, and even with personality traits. Thus, carrying the D4.7 variant has been linked to attention deficit hyperactivity disorder (ADHD), major depressive disorder, gambling, drug dependence, smoking, alcoholism, and Parkinson’s disease (Chen et al. 2011; Kaplan et al. 2008; Faraone et al. 1999; Manki et al. 1996; Ricketts et al. 1998; Laucht et al. 2005). Several studies suggested that the longer allele variants, with 7 and more repeats, are connected to specific behavioural phenotypes, including novelty seeking and risky behaviour, impulsivity, limited emotional feedback, but effective problem solving (Hohmann et al. 2009; Dmitrieva et al. 2011; Ebstein et al. 1996). Remarkably, schizophrenic patients with the shorter alleles (D4.2 and D4.4) manifest a higher therapeutic response to typical antipsychotic drugs (APDs) compared to those with D4.7 (Cohen et al. 1999). *DRD4* genes with 7-repeat alleles showed lower affinity to dopamine than D4.2 and D4.4 variants, which might explain the abovementioned differences in behavioural and neurological manifestations (Ding et al. 2002).

In recent years, several selective ligands have been developed and employed to examine DRD4 as a potential target in treating psychiatric disorders. The involvement of DRD4 in reward-related behaviours was demonstrated in a gambling model and the conditioned place preference (CPP) paradigm. In a rodent slot machine task, DRD4 agonists increased the rate of mistakes made by animals, while an antagonist restored this deficit (Cocker et al. 2014, 2016). Male and female *DRD4* knock-out mice have been observed to fail in extinction and reinstatement, but not the acquisition of cocaine-seeking behaviour in the CPP test (Ananth et al. 2019). Thanos et al. (2010) found a DRD4 dependent response to methylphenidate, amphetamine, and cocaine CPP in knock-out animals. Moreover, DRD4 were shown to play a crucial role in fear extinction processing in rats. An antagonist, L-741741, injected into the infralimbic cortex, caused an impairment in the consolidation of fear extinction memory (Pfeiffer and Fendt 2006).

Activation of DRD4, particularly enriched in the prefrontal cortex and limbic system, has been considered to benefit attention-deficit/hyperactivity disorder (ADHD) treatment by dopamine-enhancing virtue. Studies showed comparable effects of DRD4 agonist, A-412997, with a first-line therapy drug methylphenidate (Woolley et al. 2008). Other research demonstrated A-412997-induced intensifying in gamma oscillations in rats that may improve perception, attention, and working memory in autistic or schizophrenic patients (Kocsis et al. 2014).

Experimental data have supported an engagement of DRD4 signalling pathways not only in cognitive but also in emotional and stress-related functioning. Multiple research has noted that the DRD4 affinity of atypical APDs might determine their superior therapeutic efficacy and less prominent side effects in clinical practice (Newman-Tancredi et al. 2008). Although most of these medications antagonize DRD4 in the brain, others, to the contrary, act as partial agonists, such as bifeprunox, SLV313, and F15063 (Kongsamut et al. 1996; Ishiyama et al. 2007; Vangveravong et al. 2011). Some authors have even hypothesized DRD4 blockage as an independent mechanism of antipsychotic action. However, a series of clinical studies in schizophrenic patients revealed therapeutic inefficacy of several highly specific antagonists (Kramer et al 1997; Bristow et al. 1997; Corrigan et al. 2004). Similarly, inhibition of DRD4 has shown no influence on the anxiety levels of mice in the elevated plus maze (EPM) and open field (OF) tests (Navarro et al. 2003; Dulawa et al. 1999). Notably, the agonists, PD168077 and CP226269, have exhibited no antidepressant potential in the rat forced swim test (FST) (Navarro et al. 2003; Cao and Rodgers 1997).

Thus, the role of DRD4 has been revealed in a number of pathological processes in the brain, contributing for the receptors to become a promising target in pharmacotherapy of many psychiatric disorders. With beneficial impact of DRD4 stimulation on cognitive functions in animal models, the effects on emotional behaviour remain controversial. The present study, therefore, sought to investigate the influence of DRD4 activation on various behaviours in rodent models. For this goal, we used the selective DRD4 agonist, APH199, which was synthetised previously showing a favourable binding profile, strong receptor occupation, and an activation of G protein over the β-arrestin signalling pathway (Pirzer et al. 2019).

## Experimental procedures

### Animals

For our study, we used male C57BL/6 J mice (*N* = 121) (Charles River, Germany, 8–10 weeks old) and male Sprague–Dawley (SD) rats (*N* = 48) (Charles River, Germany, 8–9 weeks old). Male animals were chosen in order to avoid the possible hormonal impact on behaviour during different phases of oestrus cycle of females. Animals were grouped-housed (five and four animals per cage, for mice and rats accordingly) with food and water access ad libitum, in a temperature (22 ± 2 °C) and humidity (55 ± 10%) controlled room under a normal 12 h light–dark cycle (light on from 07:00) for rats and a reversed 12 h light–dark cycle (light on from 19:00) for mice. Experiments were carried out during the dark phase for mice and the light phase for rats.

All experiments were conducted according to the requirements of the National Institutes of Health for the humane treatment of animals and the European Communities Council Directive (86/609/EEC) and after the approval by the local government commission for animal health (Regierung von Unterfranken).

### Drug preparation

Previously we synthesized the compound APH199, which showed a high affinity for the DRD4.4 (K_i_ = 0.25 nM) and binding ratios between D2-like receptor subtypes of D2L/D4.4 = 320 and D3/D4.4 = 710 in competition binding assays. The assay measuring G protein mediated signaling in HEK cells demonstrated a bias towards G protein activation over β-arrestin recruitment with a factor of 4.9 for the ligand. Together these findings suggested the most favorable binding profile of APH199 among the recently developed DRD4 selective agonists (Pirzer et al. 2019). APH199 was dissolved in 20% Tween-80 and sterile 0.9% saline (SAL). Four doses—0.01, 0.1, 1, and 5 mg/kg were prepared in aliquots and then frozen until use -20 °C. The vehicle (VEH) was formulated by mixing 20% Tween-80 and SAL. In the Experiment II and III, mice received injections of alcohol solution at the concentration of 2 g/kg of ethanol dissolved in sterile SAL. In the experiments with rats, we used dextroamphetamine (AMPH, Fagron) with was dissolved in sterile SAL. Nine doses of AMPH (1, 2, 3, 4, 5, 6, 7, 8, 9, and 1.5 mg/kg) were prepared in aliquots and then frozen until use at -20 °C. During the “sensitization” phase, control animals were injected with SAL instead of AMPH. All drugs were injected intraperitoneally (i.p.) in the volume of 10 ml/kg for mice and 1 ml/kg for rats.

### Experiment I. Effects of APH199 on spontaneous behaviour in mice

Male C57BL/6 J mice (*N* = 15) were acclimatized to the experimental environment and handled every day for one week after arrival. During the “Habituation phase”, animals were administered with VEH and immediately after that placed in an Open Field (OF), a square grey acrylic arena with a white floor (50 × 50 × 50 cm) for one hour. All mice were tested twice in OF at a two-day interval during the “Habituation phase”. On the fourth day after the second habituation, the “Test phase” took place, including five trials with two- or three-day intertrial intervals. Mice were injected with one of five doses of APH199 according to a Latin Square design: 0, 0.01, 0.1, 1, or 5 mg/kg, i.e. each mouse received each dose during the “Test phase”. Mice were placed immediately in the OF after the injection facing a corner of the walls.

The illumination in the centre of OF arena was 20 lx. Behaviour was recorded and analysed using Biobserve Viewer III (Biobserve GmbH, Germany) or manually using videotapes. The area of 25 × 25 cm in the centre of OF arena was determined as the “central zone”, and the external part of this zone was defined as the “peripheral zone” (Müller et al. 2017; Mielenz et al. 2018).

### Experiment II. Effects of APH199 on the establishment of alcohol conditioned place preference in mice

The impact of APH199 on the establishment of alcohol-induced addictive behaviour was investigated in the conditioned place preference (CPP) paradigm (Huston et al. 2013). We used male C57BL/6 J mice (*N* = 34, *n* = 11–12/group) for this experiment. The boxes for CPP (TSE Systems, Bad Homburg, Germany) were made of non-transparent polyvinyl acrylic with a size of 40 × 15 × 20 cm (L × W × H). The place preference boxes included three chambers; two peripheral compartments were 17 cm in length, and the central zone was 6 cm. The floor in the big chambers was shrouded by a black rubber mat with either a smooth (left chamber) or patterned (right chamber) surface, while the central chamber had a white plastic floor without a mat. Behaviour was recorded automatically by the TSE Systems software using infrared sensors. The apparatus measured parameters for each compartment separately (Kalinichenko et al. 2021, 2022).

The experiment comprised four phases: acclimatization, baseline preference test (Bl), conditioning trials (I, II, III, IV, V, VI, VII), and three preference tests (T1, T2, T3). During the acclimatization phase, mice were handled and injected with SAL three times a week for habituation to the experimental procedures. In order to measure the basal level (Bl) of place preference in a pre-test, animals were administered with the VEH and placed in the central chamber of CPP with a free choice of moving between all three compartments for 20 min. We applied a counterbalanced study design whereby half of the animals were conditioned to their preferred chamber and another half to the non-preferred (Kalinichenko et al. 2021, 2022). Before the conditioning trial, mice were administered with either APH199 (0.1 or 0.5 mg/kg) or VEH, 20 min in advance. Thereafter, we injected mice with either SAL or alcohol immediately before placing them in one of two mat-covered CPP chambers for 5 min without access to the other compartments (Easton et al. 2013). Conditioning, with alcohol, and pseudoconditioning, with saline, were balanced within the experimental groups; whereas mice received 0.1 or 0.5 mg/kg of APH199 or VEH. The first preference test (T1) was conducted after one session of conditioning, as described for the BL test. T2 and T3 were performed after two and four conditioning sessions, respectively. All sessions of conditioning or testing phases were performed once a day for each mouse.

APH199, VEH and alcohol solutions were prepared and administered as described above (see Drug preparation). The concentration of 2 g/kg for alcohol was chosen based on the previous studies that demonstrated a prominent rewarding effect but without causing sedation (Easton et al. 2013; Kalinichenko et al. 2021, 2022). The illumination level of CPP boxes was 20 lx.

### Experiment III. Effects of APH199 on the retrieval of alcohol conditioned place preference in mice

To examine the effects of APH199 on the retrieval of an alcohol CPP, we applied the study design for Experiment II as described above, but with some minor adaptations. In this experiment, male C57BL/6 J mice (*N* = 36, *n* = 12/group) received injections with only SAL or alcohol (2 g/kg) immediately before the conditioning trial, whilst APH199 (0.5 or 5 mg/kg) or VEH (10 ml/kg, I.P.) was administered 20 min before each preference test (T1, T2, T3) (Kalinichenko et al. 2021, 2022).

### Experiment IV. Effects of APH199 on emotional behaviour in mice

The influence of APH199 on anxiety- and depression-like behaviour was investigated in a battery of tests. We used male C57BL/6 J mice (*N* = 36, *n* = 12/group) and analysed their behaviour in OF, Elevated plus maze (EPM), Light–dark box (LDB), Novelty-suppressed feeding (NSF) test, Forced swim test (FST), and Sucrose preference test (SPT). All tests were performed under dim light of 20 lx. Each mouse was exposed to each of the six behavioural tests once.

#### Open field

The OF test was conducted as described above for Experiment I, but with minor modifications. Animals were injected with APH199 (0.5 or 5 mg/kg, i.p.) or VEH 20 min before the test was started, and behaviour was recorded for 20 min using Biobserve Viewer III (Biobserve GmbH, Germany) (Müller et al. 2017; Mielenz et al. 2018). The OF was performed on Day 1 (*n* = 18) and Day 2 (*n* = 18) of Experiment IV, maintaining a counterbalance between the experimental groups.

#### Elevated plus maze

The EPM test was aimed to evaluate the effects of APH199 on anxiety levels in mice (Lister 1987). The apparatus was made from polyvinyl acrylic with black walls and a white floor and consisted of two closed (CA) and two open arms (OA) opposed to each other. The close arms were dimly lit (15–20 lx) and surrounded by walls of 15 cm in height, while the open arms were brighter illuminated (100 lx) and limited only by a low berm of 0.5 cm. The central zone of the maze between four arms was measured as 5 × 5 cm, and the size of each arm was determined as 30 × 5 cm (L × W). The floor of EPM was heightened at 50 cm above the floor of the experimental room. A mouse was injected with APH199 (0.5 or 5 mg/kg, i.p.) or vehicle 20 min before the test and then placed in the central zone facing one of the close arms. Animals explored the maze for 5 min with free access to all compartments, and their behaviour was recorded by Biobserve Viewer III (Biobserve GmbH, Germany). Risk assessment was analysed manually using videotapes and represents the number of entries in OA while at least one paw remained in CA (Müller et al. 2017; Mielenz et al. 2018).

#### Light–dark box

The LDB test was considered as an additional approach to measure the potential anxiolytic action of the test compound (Crawley and Goodwin 1980). The apparatus represented a white polyvinyl acrylic box with a size of 50 × 50 cm. The internal part of LDB was divided into two sections: the bigger “light chamber”, LC, (33 × 50 cm) illuminated brightly (20 lx), and the “dark chamber”, DC, (17 × 50 cm) which was dark (0 lx) and covered by a non-transparent lid. A door between two chambers served animals to have free access to both compartments. A mouse was injected with APH199 (0.5 or 5 mg/kg, i.p.) or vehicle 20 min before the test and then placed in the dark chamber facing the wall. Behaviour was tracked for 5 min using Biobserve Viewer III (Biobserve GmbH, Germany).

#### Novelty-suppressed feeding

To investigate the potential antidepressant properties of APH199, we conducted a series of three tests, starting with NSF. Before the test was started, we deprived animals of food for 24 h, but maintained free access to water. After that, mice were tested in the OF arena (as described above) for 20 min. Each animal was placed in a corner facing the wall, and a pellet of food was placed in the centre. We measured the latent time of eating and the distance moved before eating using Biobserve Viewer III (Biobserve GmbH, Germany) (Müller et al. 2017; Mielenz et al. 2018).

#### Forced swim test

The apparatus for FST involved a glass transparent cylinder measured 18 cm in diameter and 19 cm in height and filled with water (25 °C) up to 13 cm in depth. On day 1 each animal was placed in the cylinder for 15 min, and on day 2 – for 5 min (Porsolt et al. 1977). The behaviour was recorded by Biobserve Viewer III (Biobserve GmbH, Germany), and the following parameters were calculated manually: the latent period of the first floating episode and the total floating time on day 2 (Müller et al. 2017; Mielenz et al. 2018).

#### Sucrose preference test

One week before the test, we housed mice individually and provided them with access to two bottles of water ad libitum. Then we replaced one of the water bottles with another bottle containing 2% sucrose solution. During the next four days, we changed the position of bottles and measured the volume of water and sucrose solutions daily. Sucrose preference was calculated as a ratio between a volume of sucrose solution consumed to a volume of total solutions (water + sucrose) consumed in % (Müller et al. 2017; Mielenz et al. 2018).

### Experiment V. Effects of APH199 on psychotic-like behaviour in rats

Based on the findings that showed inefficacy of DRD4 inhibition in antipsychotic treatment (Kramer et al. 1997; Bristow et al. 1997; Corrigan et al. 2004), we focused here on the effects of DRD4 stimulation in the AMPH-induced psychotic rat model, as it was previously established and characterized (Peleg-Raibstein et al. 2006; Uzuneser et al. 2018, 2021). Male SD rats (*N* = 48), weighing 225–275 g at the beginning of the experiment, were used. In the first week, we handled and weighed animals daily to habituate them to the experimental test environment. Rats were randomly distributed into four experimental groups: three of them were sensitized with AMPH and one was sham-sensitized with SAL. During the “sensitization” phase, we induced a psychosis-like state. Rats were injected with either AMPH or SAL i.p. three times a day (9:00, 13:00, and 17:00) for six consecutive days. We applied an escalating dose regimen, starting from 1 mg/kg (injection 1) and adjusting to 8 mg/kg (injection 8) with steps of 1 mg/kg. The highest dose was maintained until the last injection on the sixth day of the “sensitization”. The control group received the same amount of SAL injections.

Three behavioural tests were performed to analyse different aspects of psychotic-like behaviour. The OF test was used to evaluate the anxiety level and basal locomotion. It was conducted the next day after the “sensitization” finished. Each rat was administered with APH199 at the dose of 1 or 5 mg/kg or with VEH i.p. 30 min before the test. The apparatus was made from a square grey acrylic arena with dimensions of 50 × 50 × 50 cm and an illumination level of 100 lx. The total area was divided into the central zone (25 × 25 cm) and the peripheral zone (surrounding the central zone). At the beginning of the test, a rat was placed in the corner of the OF arena for 20 min. Video recordings and the behavioural analysis were performed by Biobserve Viewer III (Biobserve GmbH, Germany) automatically.

AMPH-induced hyperlocomotion test has been widely considered a relevant model, which reflects the efficacy of antipsychotic drugs. The paradigm rests on a striking boost in the locomotor activity, induced by a single AMPH injection, of previously sensitized rats (Amato et al. 2020). We used the same arena as described above for OF test but with a dim light of 25–30 lx. Each animal was injected with APH199 (1 or 5 mg/kg, i.p.) or VEH 30 min before the test. Initially, each rat was placed in the corner of the OF arena for 20 min to record a baseline activity. Then animals were removed and injected with AMPH (1.5 mg/kg, i.p.) and placed back into the OF arena for 40 min. The activity was videotaped and analysed by Biobserve Viewer III (Biobserve GmbH, Germany). In this test, we also registered a number of rearings manually using video records (Uzuneser et al. 2018, 2021).

Disruption in the information processing is a commonly observed symptom both in schizophrenic patients and in animal models of schizophrenia. Prepulsee inhibition (PPI) of the acoustic startle response (ASR) is a method, which is generally used to assess antipsychotic efficacy and is based on the diminution of ASR amplitude in response to a low-intensity prepulse (pp) stimulus before exposure to a high-intensity pulse (P) stimulus. Psychosis emerges with a deficit of PPI and corresponds to the inability of the acoustic sensory system to habituate. The apparatus (TSE Systems, Bad Homburg, Germany) consisted of soundproof boxes with implanted loudspeakers, to produce the acoustic stimuli, and piezoelectric accelerometers, to measure the amplitude of ASR. After the injections with APH199 (1 or 5 mg/kg) or vehicle, four rats were placed into the restraining metal cages (27 × 9x10 cm) and then placed into TSE boxes individually. Each session was accompanied by background noise (68 dB) and started with a 2-min adaptation period followed by six P stimuli (100, 110 and 120 dB, twice for each intensity). After that, 16 pseudo-randomized trials were applied and repeated 10 times. Each trial consisted of three pp stimuli (74, 80, 86 dB), three P stimuli (100, 110 and 120 dB), nine combinations of pp + P stimuli, and a no stimulus trial. At the end of each session, six P stimuli were sounded again. The continuations of stimuli were 20 ms and 30 ms for pp and P trials, respectively. In the combination pp + P stimulus, the intra-trial delay was determined at 100 ms. The interval between trials was defined at 15 s (Davis and File 1984; Fendt and Fanselow 1999; Koch 1999; Peleg-Raibstein et al. 2006). The ASR was registered automatically by a software (TSE Systems). The PPI level was calculated manually with the formula: %PPI = 100 – [100 x (pp + P ASR amplitude) / P ASR amplitude)] (Amato et al. 2020; Uzuneser et al. 2018, 2021).

### Statistical analysis

Data are presented as means ± standard error of the mean (SEM). To analyse the data, we used IBM SPSS Statistics 21 software. One-way or two-way analysis of variance (ANOVA) for repeated measures were applied where appropriate to indicate the effects of factors and their interactions. Following a significant effect, Post Hoc analysis was performed using the LSD test. For pre-planned comparisons, two-tailed t-tests were used to reveal intergroup differences where appropriate. The level of significance for all tests was set at *p* < 0.05.

## Results

### APH199 did not affect spontaneous behaviour in mice

**Fig. 1:**
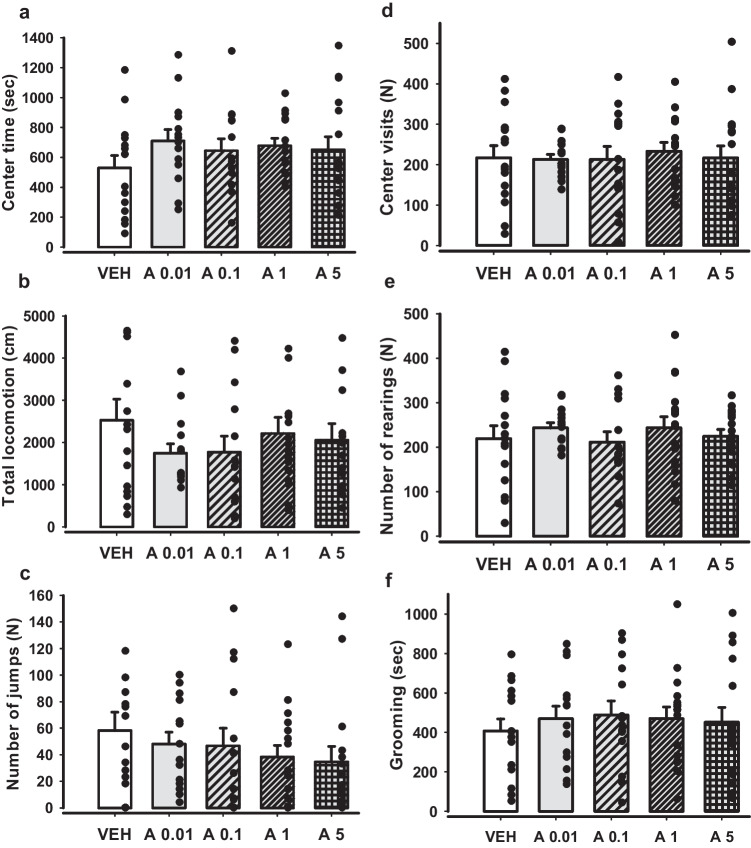
Effects of APH199 on mice behaviour in OF: **a**) total locomotion, **b**) rearing, **c**) centre time, **d**) centre visits, **e**) jumping, **f**) grooming. All mice were injected with APH199 or VEH according to the Latin square design, with the doses of 0 mg/kg (VEH) and 0.01, 0.1, 1, 5 mg/kg (APH199). Data were analysed by one-way ANOVA followed by LSD Post Hoc test (*n* = 15/per dose), no significant differences were revealed (*p* > 0.05 between all groups). VEH, vehicle; A 0.01, APH199 (0.01 mg/kg); A 0.1, APH199 (0.1 mg/kg); A 1, APH199 (1 mg/kg); A 5, APH199 (5 mg/kg)

Acute injections of APH199 at the whole range of tested doses (0.01 – 5 mg/kg) did not modify mouse behaviour in the OF during one hour of testing. In particular, no effects of APH199 were observed in center time (Fig. 1a), total locomotion (Fig. 1b), number of jumps (Fig. 1c), center visits (Fig. 1d), number of rearings (Fig. 1e), and grooming (Fig. 1f) (one-way ANOVA followed by LSD Post Hoc test, *p* > 0.05 for all parameters). Remarkably, we observed the high number of jumps for all tested animals, but with no significant differences between the groups (*p* > 0.05). We suggest, this might be associated with the repeated injections and the long duration of testing in the OF.

### No effects of DRD4 potentiation on alcohol CPP establishment and retrieval

**Fig. 2:**
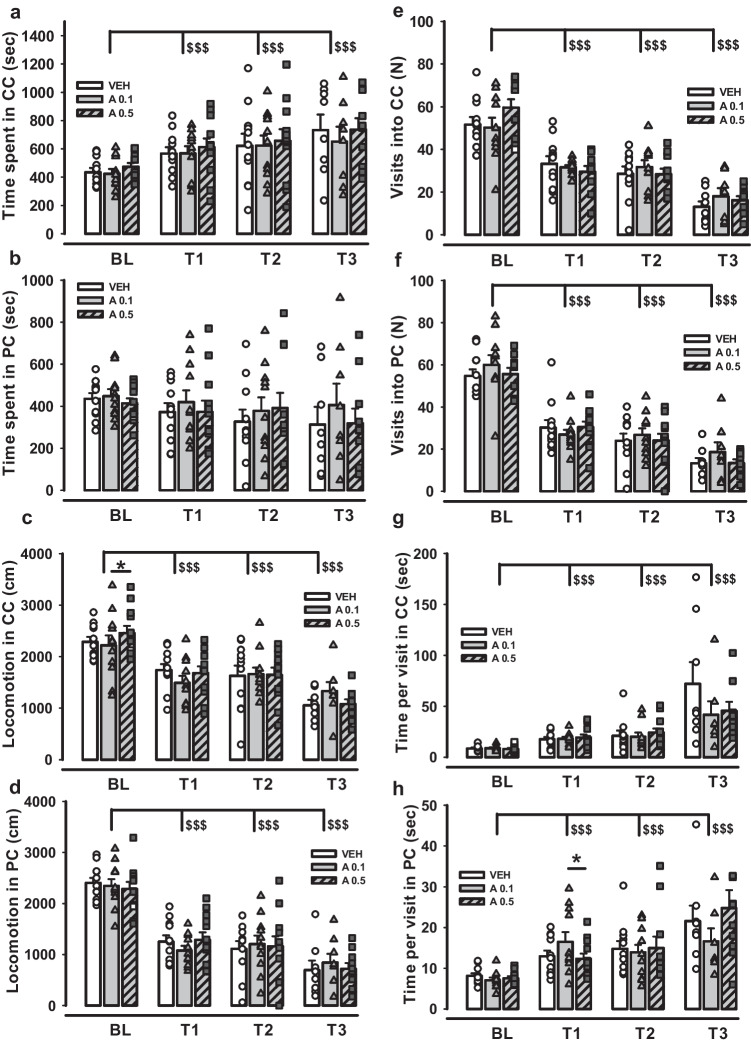
Assessment of alcohol CPP establishment in mice after APH199 or VEH treatment during conditioning trials: **a**) Time spent in CC (sec), **b**) Time spent in PC (sec), **c**) Locomotion in CC (cm), **d**) Locomotion in PC (cm), **e**) Visits into CC (N), **f**) Visits into PC (N), **g**) Time per visit in CC (sec), **h**) Time per visit in PC (sec). Data were analysed by two-way ANOVA for repeated measures followed by LSD Post Hoc test. *p < 0.05 between A0.1 and A0.5 groups, ^§§§^*p* < 0.001 vs Bl. CC, conditioned compartment; PC, pseudoconditioned compartment; VEH, vehicle; A0.1, APH199 (0.1 mg/kg); A0.5, APH199 (0.5 mg/kg); BL, baseline preference test; T1, preference test 1; T2, preference test 2; T3, preference test 1

A successful alcohol CPP was established. Mice spent more time in the conditional compartment (CC) over all three preference tests (T1, T2, T3) compared to the Bl test (two-way ANOVA for repeated measures F(3,69) = 10.669, *p* < 0.001), which is illustrated by Fig. 2a. LSD Post Hoc analysis revealed the significant differences between BL level and each of the consequent preference tests after alcohol CPP establishment (t = -3.989, *p* < 0.001, t = -3.724, *p* < 0.001, t = -5.371, *p* < 0.001, for Bl vs T1, Bl vs T2, Bl vs T3, respectively). However, no significant differences were found for the factor Group, Group*Test trial interaction and within the test trials. In the analysis of the time spent in the pseudoconditional compartment (PC), factors Test trial, Group, and Group*Test trial interaction did not play a significant role (two-way ANOVA for repeated measures followed by LSD Post Hoc test, *p* > 0.05).

Locomotion and the number of visits declined over the test trials for both CC and PC and for all experimental groups (two-way ANOVA for repeated measures; F(3,69) = 36.594, *p* < 0.001 and F(3,69) = 88.448, *p* < 0.001 for the locomotion in CC and PC, respectively; F(3,69) = 60.661, *p* < 0.001 and F(3,69) = 101.779, *p* < 0.001 for the visits made into CC and PC, respectively) (Fig. 2c-f). At the baseline level, group APH199, 0.5 mg/kg differed significantly in locomotion and visits compared to group APH199, 0.1 mg/kg (LSD, *p* = 0.035 and *p* = 0.023, respectively) (Fig. 2c). However, this intergroup variation might be accidental, as mice had not been administered with APH199 at that time point. Time per entry and locomotion per entry (Fig. 2g-h) were also analysed by two-way ANOVA for repeated measures, which demonstrated a significant effect of the factor Test trial in both CC and PC (F(3,54) = 12.655, *p* < 0.001 and F(3,66) = 13.316, *p* < 0.001 for the time per entry in CC and PC, respectively; F(3,63) = 12.507, *p* < 0.001, and F(3,66) = 6.945, *p* < 0.001 for the distance per entry in CC and PC, respectively). In PC time per entry varied significantly between groups APH199, 0.5 mg/kg and APH199, 0.1 mg/kg in the T1 trial (LSD, *p* = 0.031) (Fig. 2h), which was not further reproduced in other trials and might refer to the initial intergroup diversity.

Alcohol- and SAL-induced locomotion was also measured during seven conditioning trials. Two-way ANOVA for repeated measurements showed a significant effect by factor Group (F(2,23) = 390.531, *p* < 0.001) and Test trial (F(6,138) = 17.225, *p* < 0.001) for the locomotion after an alcohol injection. Notwithstanding, subsequent LSD tests failed to display any significant intergroup differences (*p* > 0.05). SAL-induced locomotion was affected significantly by the factor Test trial (F(6,144) = 26.039, *p* < 0.001), but not by Group and Group*Test trial interaction; LSD tests did not show differences between the groups in any trials (*p* > 0.05).

**Fig. 3:**
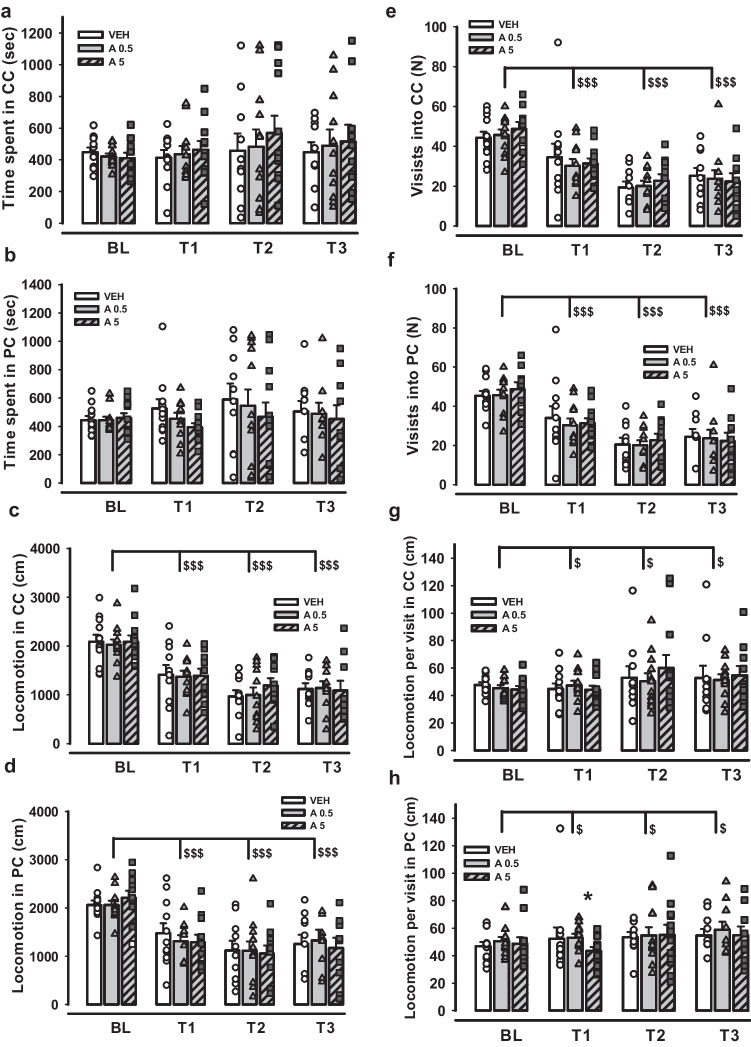
Impact of APH199 (0.5 and 5 mg/kg) or VEH administration on alcohol CPP retrieval in mice: **a**) Time spent in CC (sec), **b**) Time spent in PC (sec), **c**) Locomotion in CC (cm), **d**) Locomotion in PC (cm), **e**) Visits into CC (N), **f**) Visits into PC (N), **g**) Time per visit in CC (sec), h) Time per visit in PC (sec). Data were analysed by two-way ANOVA for repeated measures followed by LSD Post Hoc test. *p < 0.05 between A0.5 and A5 groups, ^§§§^*p* < 0.001 vs Bl. CC, conditioned compartment; PC, pseudoconditioned compartment; VEH, vehicle; A0.5, APH199 (0.5 mg/kg); A5, APH199 (5 mg/kg); BL, baseline preference test; T1, preference test 1; T2, preference test 2; T3, preference test 1

Figure 3 shows the effects of APH199, administered at doses 0.5 or 5 mg/kg, on the alcohol CPP retrieval. We observed no significant effects of factors Group, Test trial, and Group*Test trial interaction on time spent in CC or PC (two-way ANOVA for repeated measures followed by LSD Post Hoc test, *p* > 0.05) (Fig. 3a-b). The analysis showed a significant drop in locomotion in CC and PC over the test trials in comparison with Bl level (F(3,81) = 42.973, *p* < 0.001 for CC, and F(3,81) = 32.263, *p* < 0.001 for PC) (Fig. 3c-d). The number of visits into both compartments was also affected by the factor Test trial and declined significantly from Bl to T1, T2, and T3 tests (F(3,81) = 38.123, *p* < 0.001 and F(3,81) = 41.962, *p* < 0.001, in CC and PC, respectively) (Fig. 3e–f). We found a significant decrease in time and locomotion per visit (Fig. 3g-h), as well as in alcohol- and SAL-induced locomotion in the conditioning phase (not presented), in all three preference tests compared to Bl (F(3,81) = 6.468, *p* < 0.001 and F(3,81) = 10.058, *p* < 0.001 for time per visit in CC and PC, respectively; F(3,81) = 3.453, *p* = 0.02 and F(3,81) = 3.538, *p* = 0.018 for distance per visit in CC and PC, respectively; F(6,192) = 6.424, *p* < 0.001 and F(6,174) = 3.470, *p* = 0.003 for alcohol- and SAL-induced locomotion, respectively). No intergroup differences were revealed for the abovementioned parameters except locomotion per visit in PC for the groups APH199, 0.5 mg/kg and APH199, 5 mg/kg in the T1 trial (LSD, *p* = 0.021) (Fig. 3g-h). These findings suggest that DRD4 agonism affects neither the establishment nor the retrieval of an alcohol CPP.

### Negligible anxiolytic and antidepressant effects of APH199

**Fig. 4:**
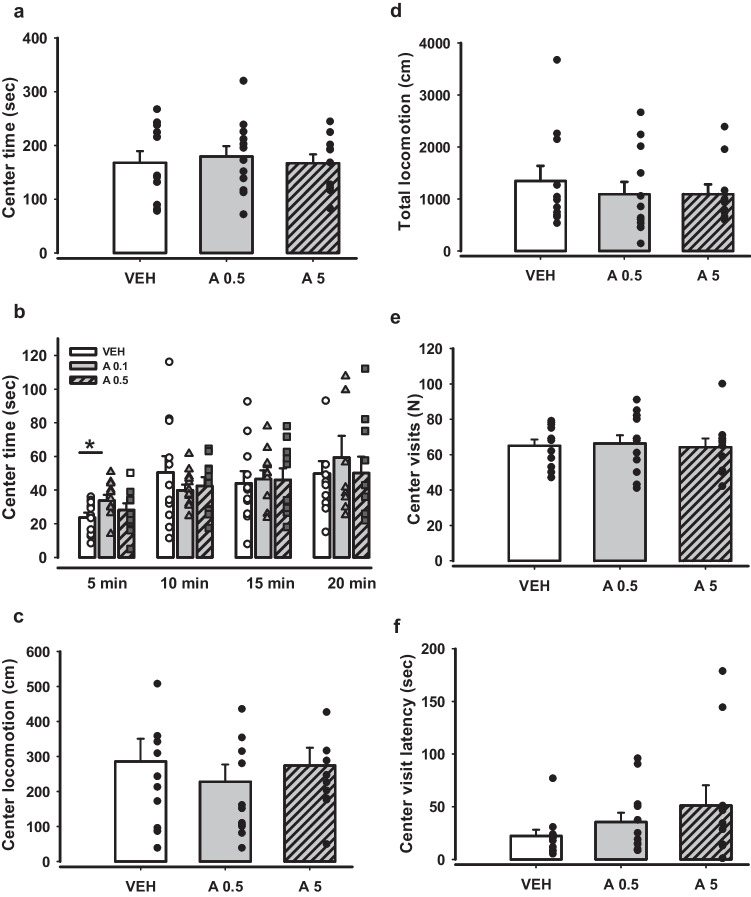
Behaviour of mice in OF after acute administration of APH199 (0.5 or 5 mg/kg) or VEH: **a**) Total center time (sec), **b**) Center time (sec) for 5 min intervals, **c**) Center locomotion (cm), d) Total locomotion (cm), **e**) Center visits (N), **f**) Center visit latency (sec). Data were analysed by two-way ANOVA for repeated measures followed by LSD Post Hoc test. *p < 0.05 between A0.5 and A5 groups. VEH, vehicle; A0.5, APH199 (0.5 mg/kg); A5, APH199 (5 mg/kg)

Anxiolytic and antidepressant properties of APH199 in two doses were analysed in several behavioural tests. Figure 4 depicts the behaviour of mice in OF test after single injections with APH199 (0.5 or 5 mg/kg) or VEH. Center time was not affected by the treatment (Fig. 4a), however, the group APH199 0.5 mg/kg spent significantly more time in the center than VEH during the first 5 min of the test (two-way ANOVA for repeated measurements F(3,90) = 8,571, *p* = 0.001 (Group), APH199, 0.5 mg/kg/VEH *p* = 0.046) (Fig. 4b). The analysis of center (Fig. 4c), total (Fig. 4d) and relative locomotion (not presented), the number of center visits (Fig. 4e) and the center visit (Fig. 4f) did not show any significant difference between the groups (two-way ANOVA for repeated measurements for 5 min intervals, and 2-tailed t-test for total testing time).

**Fig. 5:**
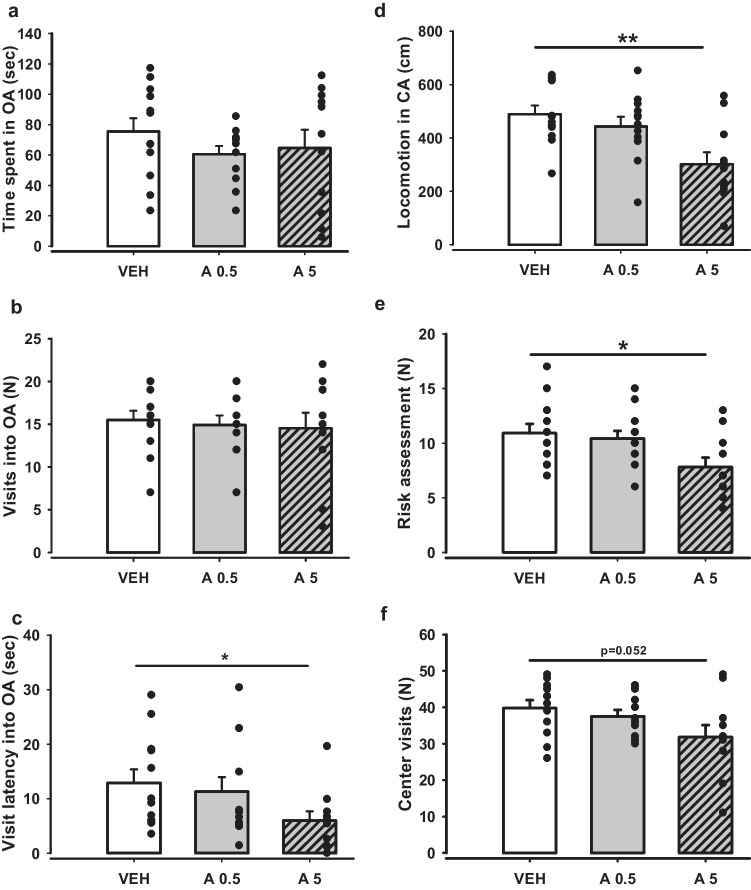
Impact of APH199 (0.5 or 5 mg/kg) or VEH on behaviour in mouse EPM test: **a**) Time spent in OA (sec), **b**) Visits into OA (N), **c**) Visit latency into OA (sec), **d**) Locomotion in CA (cm), **e**) Risk assessment (N), **f**) Center visits (N). Data were analysed by two-tailed t-test. *p < 0.05, **p < 0.01 vs VEH group. OA, open arms; CA, closed arms; VEH, vehicle; A0.5, APH199 (0.5 mg/kg); A5, APH199 (5 mg/kg)

The EPM is one of the key tests we used to assess APH199-induced modifications in an anxiety level. Two-tailed t-test analysis found no significant differences between the groups in total (Fig. 5a) and relative time spent, visits and locomotion in the OA (*p* > 0.05). However, we observed reduced locomotor activity in CA (*p* = 0.003) (Fig. 5d), visit latency into OA (*p* = 0.03) (Fig. 5c), and risk assessment behaviour (*p* = 0.019) (Fig. 5e) for the mice injected with 5 mg/kg of APH199. The same group of animals demonstrated a strong tendency for a decline in center visits (*p* = 0.052) (Fig. 5f).

**Fig. 6:**
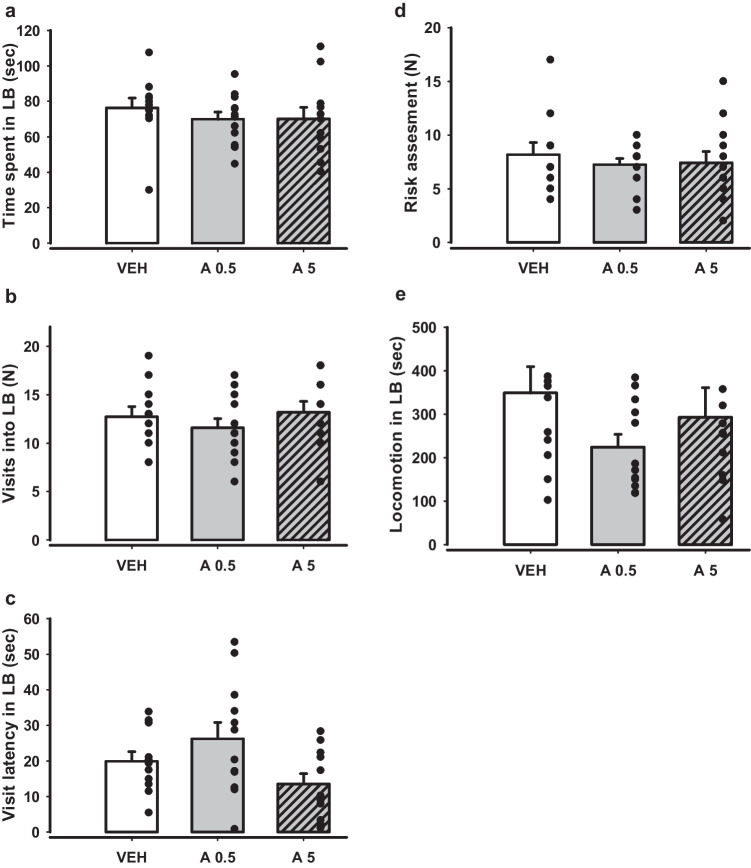
Effects of APH199 (0.5 or 5 mg/kg) or VEH on mice behaviour in LDB: **a**) Time spent in LB (sec), **b**) Visits into LB (N), **c**) Visit latency in LB (sec), **d**) Risk assessment (N), **e**) Locomotion in LB (cm). Data were analysed by two-tailed t-test. LB, light box; VEH, vehicle; A0.5, APH199 (0.5 mg/kg); A5, APH199 (5 mg/kg)

Potential anxiolytic-like effects were further analysed in the LDB test after administration of APH199 (0.5 or 5 mg/kg) or VEH (Fig. 6). Statistical analysis did not reveal any significant intergroup differences for all the measured parameters: total (Fig. 6a) and relative time spent, locomotion (Fig. 6e), visits made (Fig. 6b), visit latency (Fig. 6c), and risk assessment in LB (Fig. 6d). The relatively small anxiolytic effects, which were observed in EPM test for the group received APH199, 5 mg/kg, were not reproduced in LDB and, therefore, may not be considered as a reliable outcome of DRD4 stimulation.

**Fig. 7:**
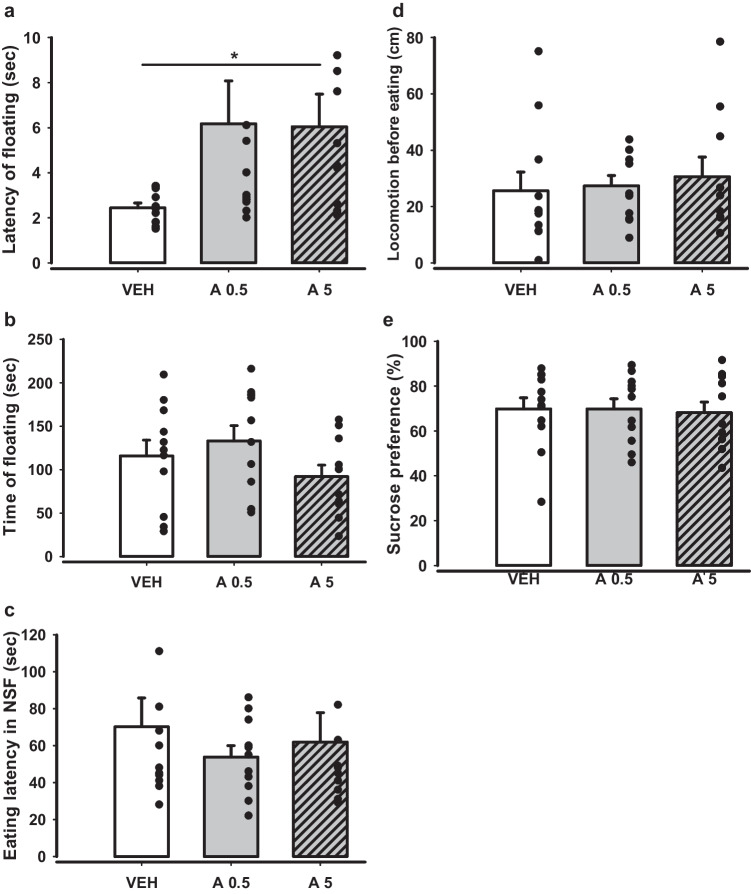
Behaviour in FST, NSF, and SPT of mice after APH199 (0.5 or 5 mg/kg) or VEH administration: **a**) Latency of floating (sec) in FST, **b**) Time of floating (sec) in FST, **c**) Eating latency (sec) in NSF test, **d**) Locomotion before eating (cm) in NSF teats, **e**) Sucrose preference (%) in SPT. Data were analysed by two-tailed t-test. *p < 0.05 vs VEH group. FST, forced swim test; NSF, novelty suppressed feeding; SPT, sucrose preference test; VEH, vehicle; A0.5, APH199 (0.5 mg/kg); A5, APH199 (5 mg/kg)

Subsequent three approaches were aimed to analyse the impact of the DRD4 agonist on depression-like and anhedonic behaviour and included FST, NSF, and SPT (Fig. 7). Experimental groups did not differ significantly in eating latency and locomotion before eating in the NSF test (Fig. 7c-d), along with sucrose preference in SPT (Fig. 7e). FST demonstrated no effects of treatment in a key parameter, time of floating (Fig. 7b). However, the latency of floating was longer for group APH199, 5 mg/kg compared to VEH (p = 0.032) (Fig. 7a). This may suggest a mild antidepressant effect.

Thus, the battery of tests, which was designed to measure anxiety and depression-like behaviour, indicated sporadic anxiolytic and antidepressant effects of DRD4 agonism.

### Exacerbation of psychotic-like symptoms after APH199 administration in a dose-dependent manner

**Fig. 8:**
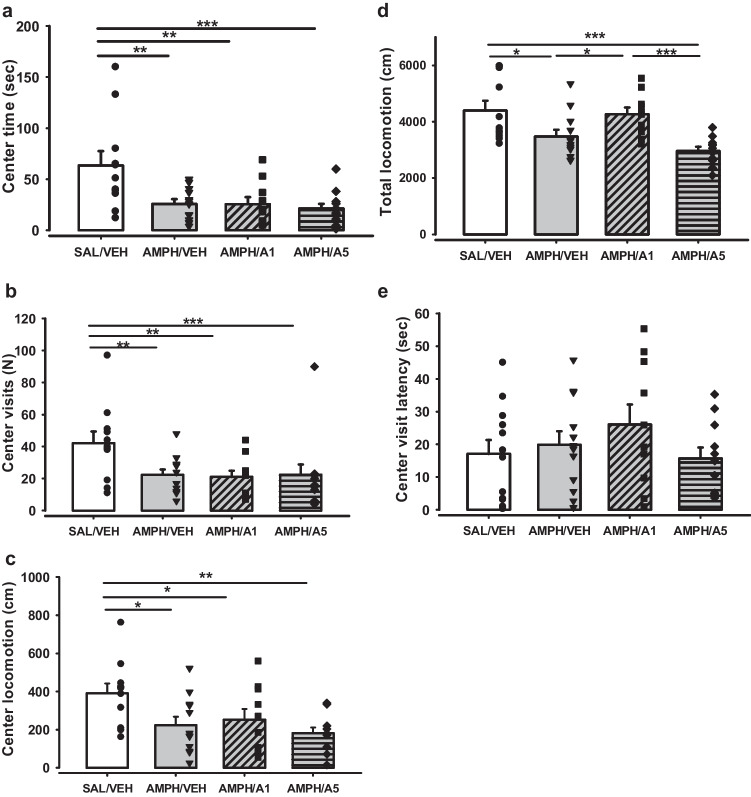
Behaviour of rats in the OF test after single injections of APH199 (1 or 5 mg/kg) or VEH: **a**) Center time (sec) for 5-min intervals, **b**) Center time (sec) for 20-min testing, **c**) Center visits (N), **d**) Center locomotion (cm), **e**) Total locomotion (cm), **f**) Center visit latency (sec). Data were analysed by the one-way ANOVA followed by LSD Post Hoc test. *p < 0.05, **p < 0.01, ***p < 0.001 between groups. SAL/VEH, SAL-sensitized and VEH-treated group; AMPH/VEH, AMPH-sensitized and VEH-treated group; AMPH/A1, AMPH-sensitized and APH199 (1 mg/kg)-treated group; AMPH/A5, AMPH-sensitized and APH199 (5 mg/kg)-treated group

We examined the anxiety level and locomotor activity of psychotic-like rats in the OF test after single injections of APH199 at the dose of 1 or 5 mg/kg or VEH. The one-way ANOVA pointed out a significant drop in the center time for all three AMPH-sensitized groups in comparison with a control (factor Group F(3,40) = 6.366, *p* < 0.001; followed by LSD Post Hoc test for Multiple comparisons *p* = 0.002, *p* = 0.003, *p* < 0.001, SAL/VEH vs AMPH/VEH, AMPH/APH199, 1 mg/kg, and AMPH/APH199, 5 mg/kg, respectively) (Fig. 8a). The same effects of AMPH sensitization were observed for the center visits (factor Group F(3,40) = 5.388, *p* = 0.003; followed by LSD *p* = 0.005, *p* = 0.004, *p* < 0.001, SAL/VEH vs AMPH/VEH, AMPH/APH199, 1 mg/kg, and AMPH/APH199, 5 mg/kg, respectively) and center locomotion (factor Group F(3,40) = 3.921, *p* = 0.015; followed by LSD Post Hoc test p = 0.012, *p* = 0.044, *p * = 0.002, SAL/VEH vs AMPH/VEH, AMPH/APH199, 1 mg/kg, and AMPH/APH199, 5 mg/kg, respectively) (Fig. 8b-c). The analysis of total locomotion showed a significant difference between SAL/VEH group vs AMPH/VEH (*p * = 0.011) and AMPH/APH199 5 mg/kg (*p* < 0.001), along with AMPH/APH199, 1 mg/kg vs AMPH/VEH (*p* = 0.033) and AMPH/APH199, 5 mg/kg (*p * < 0.001). No significant intergroup variations were found for the central visit latency. Thus, all investigated parameters declined over 20 min of testing for all experimental groups suggesting habituation of animals. Psychotic phenotype affected significantly central time, central visits, and central locomotor activity. AMPH-sensitized rats showed a lower level of locomotion compared to the control group; however, APH199 treatment at the dose of 1 mg/kg restored this drop.

**Fig. 9:**
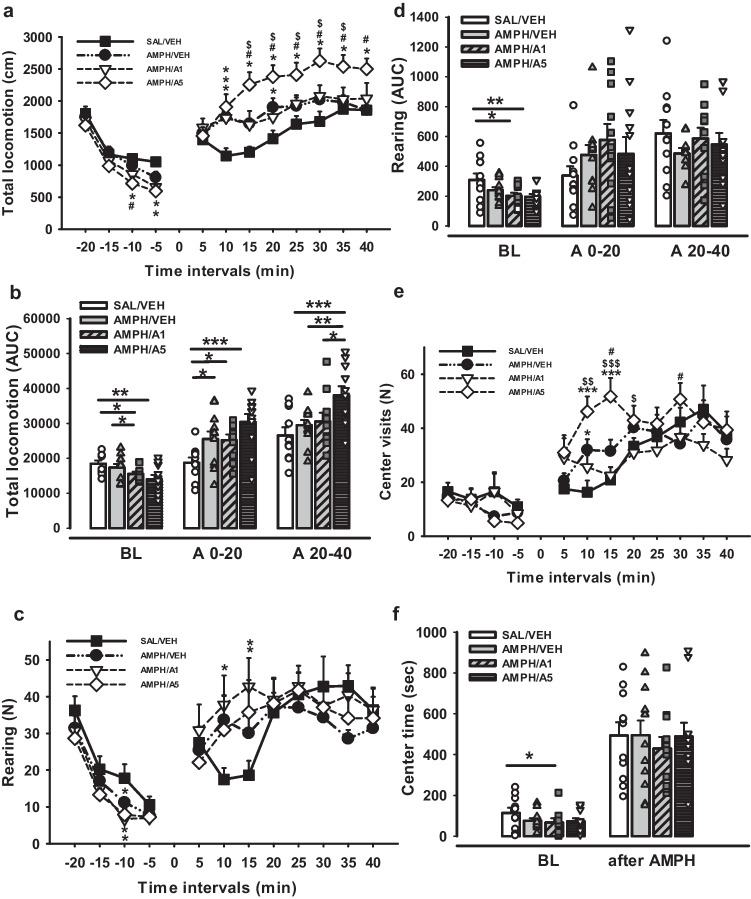
Effects of APH199 on psychotic behaviour in rats: **a**) Total locomotion (cm) in 5-min time intervals, **b**) Total locomotion (AUC) for the Bl, and the first and second 20-min intervals after AMPH challenge (1.5 mg/kg), **c**) Rearing (N) in 5-min time intervals, **d**) Rearing (AUC) for the Bl, and the first and second 20-min intervals after AMPH challenge (1.5 mg/kg), **e**) Center visits (N) in 5-min time intervals, f) Center time (sec) for Bl interval and 40 min after AMPH challenge (1.5 mg/kg). Data were analysed by the one-way ANOVA followed by LSD Post Hoc test. *p < 0.05, **p < 0.01, ***p < 0.001 between groups, ^#^*p* < 0.05 vs AMPH/VEH group, ^$^*p* < 0.05, ^$$^*p* < 0.01, ^$$$^*p* < 0.001 vs AMPH/A1. SAL/VEH, SAL-sensitized and VEH-treated group; AMPH/VEH, AMPH-sensitized and VEH-treated group; AMPH/A1, AMPH-sensitized and APH199 (1 mg/kg)-treated group; AMPH/A5, AMPH-sensitized and APH199 (5 mg/kg)-treated group (AUC—area under the curve)

In order to examine the effects of DRD4 activation on psychotic-like behaviour, the AIH test was conducted (Fig. 9). We evaluated total locomotion, measured as total distance moved (cm), during 5-min intervals and during three time intervals: 20 min of BL, 0–20 min (AI0-20) and 20–40 min (AI20-40) after AMPH injection (Fig. 9a-b). AUC analysis of changes in locomotion over time showed that it was affected significantly by the factor Group (one-way ANOVA F(3,39) = 3.933, *p* = 0.015; F(3,39) = 5.588, *p* = 0.003; F(3,39) = 4.823, *p* = 0.006, for BL, AI0-20 and AI20-40, respectively). LSD Post Hoc test for Multiple comparisons indicated that during Bl interval AMPH/ APH199, 1 mg/kg and AMPH/APH199, 5 mg/kg groups moved less compared to SAL/VEH (*p* = 0.049 and *p* = 0.003, respectively); whereas the AMPH/APH199, 5 mg/kg group showed even lower locomotion than AMPH/VEH animals (*p* = 0.018). After the injection of AMPH, previously AMPH-sensitized groups demonstrated significantly higher locomotion in comparison with control rats (*p* = 0.021, *p* = 0.034, *p* < 0.001, for AMPH/VEH, AMPH/APH199, 1 mg/kg, AMPH/APH199, 5 mg/kg vs SAL/VEH, respectively). During AI20-40, only the AMPH/APH199, 5 mg/kg group differed significantly from the other three groups reaching the highest locomotion level in the test (*p* < 0.001, *p* = 0.008, *p* = 0.023, for SAL/VEH, AMPH/VEH, AMPH/APH199, 1 mg/kg vs AMPH/APH199, 5 mg/kg, respectively).

Rearing behaviour manifests either vertical locomotor activity or exploratory behaviour and was measured as a number of free and wall rearings. Previously, it was shown that a raise in rearing activity was observed in AMPH-sensitized rats and can be considered one of the psychotic symptoms (Uzuneser et al. 2018). In this experiment, we analysed the AUC of rearing for the same time intervals as for total locomotion: BL, AI0-20 and AI20-40 (Fig. 9d). One-way ANOVA followed by LSD Post Hoc test indicated the significant differences during Bl between SAL/VEH vs AMPH/APH199, 1 mg/kg and AMPH/APH199, 5 mg/kg groups (*p* = 0.013 and *p* = 0.007, respectively). Further analysis of rearing for 5-min time intervals during the total testing time revealed a significant boost after 15 min of AMPH injection for AMPH/APH199, 1 mg/kg and AMPH/APH199, 5 mg/kg compared to SAL/VEH (one-way ANOVA, *p* = 0.015 and *p *= 0.048, respectively) (Fig. 9c).

Over the Bl period, rats administered with APH199, 1 mg/kg spent significantly less time in the central zone than control animals SAL/VEH (one-way ANOVA followed by LSD Post Hoc test, *p* = 0.022), although those differences were withdrawn after the AMPH challenge (Fig. 9f). AMPH/APH199, 5 mg/kg group made a lower number of central visits at the Bl level (*p * = 0.042), whereas it became diametrically opposed after AMPH injection (*p* = 0.041 vs SAL/VEH, *p *= 0.008 vs AMPH/APH199, 1 mg/kg). One-way ANOVA for repeated measurements demonstrated significant differences in the central visits between SAL/VEH vs AMPH/VEH at 10 min after the AMPH challenge (*p* = 0.015); AMPH/APH199, 5 mg/kg vs SAL/VEH and AMPH/APH199, 1 mg/kg at 10 min (*p* < 0.001 and p = 0.009, respectively); AMPH/APH199, 5 mg/kg vs SAL/VEH, AMPH/VEH and AMPH/APH199, 1 mg/kg at 15 min (*p* < 0.001, *p * = 0.013, and *p *< 0.001, respectively); AMPH/APH199, 5 mg/kg vs AMPH/APH199, 1 mg/kg at 20 min (*p* = 0.039); AMPH/APH199, 5 mg/kg vs AMPH/VEH at 30 min (*p *= 0.019) (Fig. 9e). APH199 administration and AMPH sensitization had no effect on latency of the first center visit over Bl (20 min) or AMPH challenge (40 min) periods (*p* > 0.05).

Altogether, the results indicated a significant enhancement of total locomotion and rearing in response to AMPH sensitization. Psychotic-like effects were aggravated after DRD4 activation with APH199 at 5 mg/kg, as demonstrated by the increased total distance moved, the number of rearings, and the central visits.

**Fig. 10:**
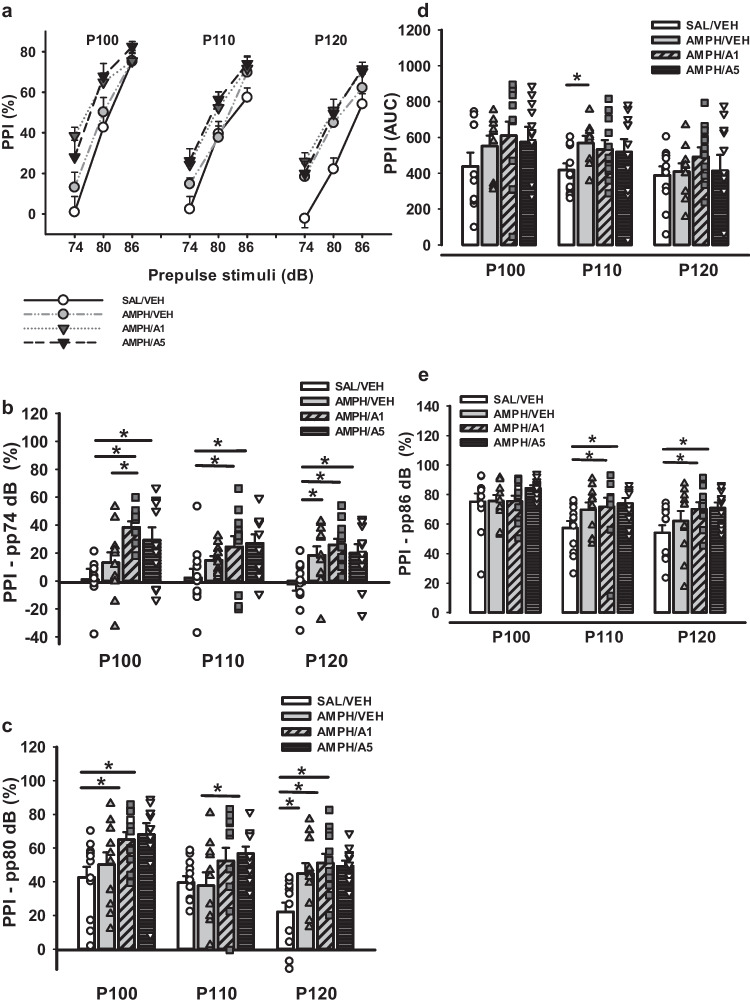
Prepulsee inhibition of acoustic startle response after APH199 or VEH administration in rats: **a**) PPI (%) for combinations of pp + P stimuli, **b**) PPI for pp74 dB (%), **c**) PPI for pp80 dB (%), **d**) PPI (AUC) for P100, P110, and P120 stimuli, **e**) PPI for pp86 dB (%). Data were analysed by two-way ANOVA for repeated measures followed by LSD Post Hoc test. *p < 0.05 between groups. PPI, prepulse inhibition; SAL/VEH, SAL-sensitized and VEH-treated group; AMPH/VEH, AMPH-sensitized and VEH-treated group; AMPH/A1, AMPH-sensitized and APH199 (1 mg/kg)-treated group; AMPH/A5, AMPH-sensitized and APH199 (5 mg/kg)-treated group (AUC—area under curve)

Analysis of PPI values for the different combinations of pp + P stimuli did not demonstrate significant differences between the groups (Fig. 10a). Two-way ANOVA for repeated measurements for AUC analysis of PPI revealed a significant increment between SAL/VEH vs AMPH/VEH groups (*p* = 0.026) and a strong tendency vs AMPH/APH199, 1 mg/kg (*p * = 0.057) in response to the P110 stimulus (Fig. 10d). To evaluate PPI specifically for each pp stimulus and its appropriate combinations with P stimuli, we carried out two-way ANOVA for repeated measurements. The analysis of PPI for pp 74 dB stimuli combinations indicated the effect of factor Group (F(11,138) = 3.994, *p * < 0.001) and significant differences between the following groups: SAL/VEH vs AMPH/APH199, 1 mg/kg (*p* < 0.001) and AMPH/APH199, 5 mg/kg (*p * = 0.002), and AMPH/VEH vs AMPH/APH199, 1 mg/kg (*p * = 0.006) for P100; SAL/VEH vs AMPH/APH199, 1 mg/kg (*p * = 0.014) and AMPH/APH199, 5 mg/kg (*p * = 0.007) for P110; SAL/VEH vs AMPH/VEH (*p * = 0.023), AMPH/APH199, 1 mg/kg (*p * = 0.002), and AMPH/APH199, 5 mg/kg (*p * = 0.014) for P120 (Fig. 10b). Two-way ANOVA for pp 80 dB showed a significant effect by factors Group (*p * < 0.001) and P stimulus (*p * = 0.002), F(11,138) = 4.392. We also measured differences between the groups for all three P stimuli: SAL/VEH vs AMPH/APH199, 1 mg/kg (*p * = 0.008) and AMPH/APH199, 5 mg/kg (*p * = 0.003), and AMPH/VEH vs AMPH/APH199, 5 mg/kg (*p * = 0.040) for P100; AMPH/APH199, 5 mg/kg vs SAL/VEH (*p * = 0.044) and AMPH/VEH (*p * = 0.029) for P110; SAL/VEH vs AMPH/VEH (*p * = 0.008), AMPH/APH199, 1 mg/kg (*p * < 0.001), and AMPH/APH199, 5 mg/kg (*p * = 0.002) for P120 (Fig. 10c). The analysis for pp 86 dB displayed the effect of factors Group and P stimulus (F(11,138) = 3.138, *p * = 0.004 and *p * < 0.001, respectively). The differences reached significance in response to P110 and P120 for SAL/VEH vs AMPH/APH199, 1 mg/kg (*p * = 0.033 and *p * = 0.017, for P110 and P120, respectively) and AMPH/APH199, 5 mg/kg (*p * = 0.016 and *p * = 0.014, for P110 and P120, respectively) (Fig. 10e).

**Fig. 11:**
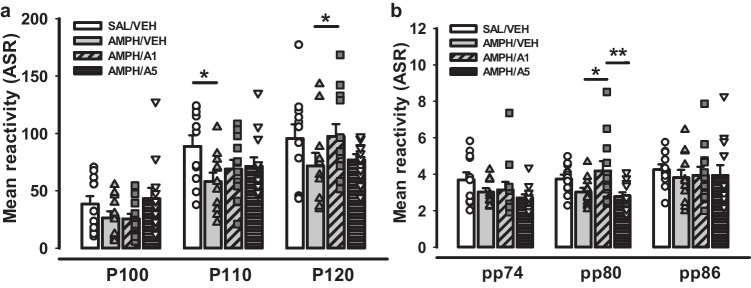
Acoustic startle response (ASR) after APH199 or VEH administration for pulse-alone and prepulse-alone acoustic stimuli in rats: **a**) (ASR) for P100, P110, and P120 dB stimuli, **b**) Mean reactivity (ASR) for pp74, pp80, pp86 dB prepulse stimuli. Data were analysed by two-way ANOVA for repeated measures followed by LSD Post Hoc test. *p  < 0.05, **p  < 0.01 between groups. ASR, acoustic startle response; SAL/VEH, SAL-sensitized and VEH-treated group; AMPH/VEH, AMPH-sensitized and VEH-treated group; AMPH/A1, AMPH-sensitized and APH199 (1 mg/kg)-treated group; AMPH/A5, AMPH-sensitized and APH199 (5 mg/kg)-treated group

ASR for pulse- and prepulse-alone stimuli were analysed with two-way ANOVA for repeated measurements followed by the LSD Post Hoc test. Figure 11a depicts the amplitude of ASR to 100, 110, or 120 dB pulse alone stimuli as mean values over 10 trials for each group. The analysis revealed significant effects by factors Group (F(3;138) = 3.219, *p * = 0.025) and pulse stimulus (F(2;138) = 40.124, *p * < 0.001). In response to P110 acoustic stimulus, SAL/VEH rats displayed higher ASR levels than AMPH/VEH group (*p * = 0.014), while ASR to P120 displayed a significant difference between AMPH/VEH and AMPH/APH199, 1 mg/kg groups (*p * = 0.035). These data suggest that the AMPH sensitization evoked a deficit in ASR for all pulse alone stimuli, but with a more prominent effect in response to P110.

Data analysis of ASR to the prepulse-alone stimuli showed significant effects by factors Group (F(3;138) = 2.743, *p * = 0.046) and prepulse stimulus (F(2;138) = 5.144, *p * = 0.007) (Fig. 11b). Visual inspection of the data suggests that AMPH-sensitization reduced prepulse ASR. This effect was partly reversed by APH199. Animals from the group AMPH/APH199, 1 mg/kg, manifested the higher ASR amplitude to pp80 stimulus compared to AMPH/APH199, 5 mg/kg (*p * = 0.008) and AMPH/VEH (*p * = 0.031).

These results suggest that the rather paradoxical increase in PPI levels after AMPH-sensitization, with and without APH199 treatment, may have been caused mainly by distinct effects on ASR amplitudes to prepulse- and pulse-alone stimuli, rather than by effects on stimulus gating.

## Discussion

We investigated the effects of DRD4 stimulation in a wide range of rodent behavioural tasks. For this goal, we used a recently developed highly selective ligand, APH199, with a promising receptor binding profile and cellular signalling pathway (Pirzer et al. 2019). Several approaches were employed to elicit behavioural outcomes in the animal models of alcohol addiction, anxiety, depression, and schizophrenia. To our knowledge, this study is the first to investigate APH199 in vivo and to demonstrate the behavioural effects of DRD4 activation in a rat model of AMPH-induced psychosis.

Our results indicated that acute administration of APH199 exacerbated the consequences of AMPH sensitization in a dose-dependent manner in rats. AIH and PPI tests are accepted widely to predict the therapeutic efficacy of APDs in preclinical studies (Amato et al. 2020; Gobira et al. 2013; Peleg-Raibstein et al. 2012), and, therefore, were chosen to examine the effects of DRD4 potentiation, along with the OF test. In our experiments, AMPH-sensitized rats may developed distinctive “psychotic-like” symptoms, resembling positive symptoms, such as agitation and hyperactivity, in schizophrenic patients (Angrist et al. 1970), through the enhanced locomotor and rearing activity, time spent in the center of OF and a decline in a startle response amplitude. The previous findings corroborated these effects (Uzuneser et al. 2018, 2021), which arise from increased dopaminergic activity in the mesolimbic pathway after AMPH stimulation (Iversen 1987). The inhibition of dopamine D2 receptors with APDs normally reverses the behavioural disruptions (Peleg-Raibstein et al. 2012; Gobira et al. 2013; Amato et al. 2011; Möller et al. 2017). In the AIH test, the ligand APH199 at the higher dose (5 mg/kg) evoked a dramatic boost in locomotion levels after the AMPH challenge in comparison with control and AMPH-sensitized but VEH-treated animals. The lower dose of APH199 (1 mg/kg), induced an enhanced rearing number in the first 20 min after AMPH injection. These data suggest that stimulation of DRD4 may enhance significantly the dopamine levels not only in the mesocortical pathways but also in the mesolimbic structures, which are responsible for the pathophysiology of psychosis (Vallone et al. 2000). Remarkably, during the period before the challenge, we observed a decline in both locomotion and rearing for the AMPH-sensitized groups compared to SAL-pretreated rats. These results are in line with our data from the OF test, where we found the same group-specific effects. The decreased locomotor activity in AMPH-sensitized animals might be associated with the effects of AMPH withdrawal, and administration regimen and withdrawal period were shown to play a significant role. Similarly, the locomotor depression was observed after 24 h of AMPH withdrawal in rats treated with an escalating dose procedure (1–10 mg/kg/d) but not after 5 days or different administration schedule (Russig et al. 2005). The doses of AMPH may also affect the spontaneous locomotor activity. Animals treated with 5 mg/kg/d AMPH for 8 consecutive days showed the reduction in locomotion on withdrawal day one; but no such effect was found in 0.5 and 2.5 mg/kg/d treated rats (Schreiber et al. 1976). The shorter time spending in the central zone both in OF and AIH tests (before AMPH challenge) might also suggest higher anxiety for the AMPH-treated animals. In methamphetamine users, research showed comorbidity of anxiety along with other drug-induced effects, such as psychosis, agitation, and paranoia (Paulus and Stewart 2020), that may explain the observed anxiogenic action of AMPH in our experiments. Interestingly, in the OF test, both in mice and rats, APH199 administration did not escalate locomotion. The booster effect of DRD4 stimulation occurred only after the AMPH challenge; it designates the synergistic exacerbation of psychotic constituents, but not only motor hyper functioning.

The analysis of sensorimotor gating revealed unexpected effects on percentage PPI and startle response in the AMPH-sensitized groups. As it was previously reported (Uzuneser et al. 2018, 2021), a reduction of PPI is a hallmark of a psychotic-like state of animals pretreated with AMPH in escalating-dose regimen. However, in this study we observed no difference or even an enhanced percentage PPI in AMPH and APH199 administered groups when comparing with control rats. Interestingly, we found a decline in startle response for both, prepulse- and pulse-alone stimuli, after AMPH sensitization. According to the formula we used for PPI calculation, the amplitude of ASR and percentage PPI are in inverse correlation. In view of this, we suggest that AMPH-pretreated animals developed high sensitivity to the acoustic stimuli or as a consequence of higher anxiety to a novel environment. Moreover, the effects of AMPH on PPI might be dose-, schedule- and context dependent. Russig et al. (2005) found that only an escalating dose regimen of AMPH administration at the doses 1–10 mg/kg/d evoked a reduction of PPI, while intermittent and escalating dose (1–5 mg/kg/d) schedules did not led to a disruption of PPI. Similar to our results, this study showed a decrease in an amplitude of a startle response in AMPH-sensitized (escalating dose schedule, 1–5 mg/kg/d) animals compared to control groups. Other studies have revealed the impact of DA agonists administration paired with PPI testing on a decline of percentage PPI (Martin-Iverson 1999; Zhang et al. 1998). Taken together, we suggest that despite the presence of robust behavioral sensitization, the observed effects on PPI might be associated with the specific schedule and doses of administration, and/or the context of testing environment. Thus, the effects of APH199 in this paradigm may only be interpreted as effects on pulse- and prepulse sensitivity alone.

Van Tol and colleagues (1991) hypothesized that the outstanding therapeutic action of atypical APDs is associated particularly with the inhibition of DRD4. Nevertheless, several clinical trials failed to prove the efficacy of DRD4 antagonists, pointing out against their implementation in the treatment of schizophrenic disorders (Kramer et al. 1997; Bristow et al. 1997; Corrigan et al. 2004). Even though most APDs are highly effective against positive symptoms, only a few of the atypical APDs, such as clozapine, risperidone, olanzapine and lurasidone, have shown improvement in domains of cognitive functions (Harvey and Davidson 2002; Meltzer et al. 2003). DRD4 ligands though have been proposed to be applied in the challenging treatment of cognitive impairment associated with schizophrenia (CIAS) (Meltzer 2015). Several antagonists and agonists were investigated in animal models of CIAS. Research surprisingly indicated the beneficial effects for both groups of ligands in cognitive and memory tasks. In monkeys, antagonists reversed stress-induced working-memory deficit in the delayed response task (Arnsten et al. 2000), and improved phencyclidine-induced cognitive deficits in the object retrieval task (Jentsch et al. 1999). In accordance with these findings, DRD4 agonists facilitated memory functions in the novel object and social recognition tests, restored a phencyclidine-induced cognitive deterioration and avoidance learning response (Bernaerts and Tirelli 2003; Woolley et al. 2008; Sood et al. 2011; Browman et al. 2005). Researchers suggested, therefore, that a moderate level of DRD4 potentiation might be beneficial in the treatment of CIAS and may improve the efficacy of APDs in a broader spectrum of schizophrenic symptoms (Newman-Tancredi et al. 2008). However, in light of our findings, the development of antipsychotic compounds with additional DRD4 agonistic binding profiles may worsen the risk/benefits ratio for schizophrenic patients. From a clinical perspective, such drugs will probably exaggerate the patients’ symptoms which further might provoke the discontinuation of the treatment.

In the model of alcohol abuse, we demonstrated no significant impact of APH199 at any dose on the CPP establishment and retrieval. A few observed differences took place accidentally during the baseline period, before the treatment, or only in the pseudoconditioning compartment. However, a line of evidence sustained the role of DRD4 signalling in addictive behaviour. VNTR polymorphism of the DRD4 gene has been found to be associated with chronic alcoholism, smoking, cocaine and heroin dependences (Hutchison et al. 2002a, b; Shao et al. 2006). In addition, animal studies showed the beneficial impact of some DRD4 antagonists to attenuate or reverse the rewarding effects of cocaine and methamphetamine (Ukai and Mitsunaga 2005; Yan et al. 2004), morphine-induced withdrawal syndrome (Mamiya et al 2004), and nicotine reinstatement (Yan et al. 2012). On the contrary, DRD4 agonists, such as PD 168,077, have been reported to have no effect on the reinstatement of extinguished nicotine-seeking behaviour (Yan et al. 2012). Although, we assume that the effects of DRD4 modulators might be determined by the specific behavioural model, reinforcing drug or animal species; our experiments, in line with previous research, have not indicated a significant impact of the agonists on an animal model of alcohol abuse.

The present study displayed no remarkable behavioural alterations affected by DRD4 potentiation in a battery of emotional tests. Three methods measured anxiety-related behaviours and showed minor group effects for 0.5 mg/kg of APH199 in OF and 5 mg/kg of APH199 in EPM, whereas, no differences were observed in LDB. Notably, we did not find significant variations in the main behavioural parameters for all three tests, suggesting relatively small and inconsistent anxiolytic or anxiogenic properties of APH199 in our mouse models. Notwithstanding, Avila and colleagues (2020) examined another DRD4 agonist, PD-168077, and showed enhanced time spent in OA in the EPM and declined duration of burying behaviour in the test. The authors concluded an anxiolytic action of the compound. However, it was injected locally in the globus pallidus and they used rats in their experiments. The earlier research with the DRD4 antagonist, L-745870, surprisingly demonstrated the same behavioural effects: the elevated number of OA visits and OA time in the EPM test and reduced time of burying in the shock-probe test (Shah et al. 2004). In that study, L-745870 was injected intracranially in the medial prefrontal cortex of rats. Accordingly, the role of DRD4 in anxiety-related behaviours remains ambiguous and requires further research.

Antidepressant-like properties were assessed in the models of behavioural despair, hyponeophagia and anhedonia in mice. The results indicated no significant differences in key parameters for all mentioned tests; only the enhanced latency of floating in FST was observed for APH199 (5 mg/kg). Studies with two other agonists, PD 168,077 and CP 226,269, likewise revealed no meaningful effects in the rat FST (Basso et al. 2005). Experiments with the induced depression-like behaviour in rats demonstrated a correlation between the levels of DRD4 in the amygdala and floating time in FST, and between nucleus accumbens, olfactory tubercle and sucrose preference rate (Bai et al. 2017). These findings may indicate engagement of the described receptors in specific brain structures and various depressive-like patterns. Consistent with these results obtained using other DRD4 agonists, we found no substantial support for an antidepressant potential of APH199.

A number of factors limits our study. First, all our experiments were performed only in male rodents, even though sex differences may play a significant role in behavioural responses to pharmacological challenges (Kalinichenko et al. 2021, 2022). Research showed that the results of many behavioural tests, including FST, EPM, OF, amphetamine- and apomorphine-induced locomotion, varied significantly not only after pharmacological interventions, but also during baseline activity (Simpson et al. 2012; Knight et al. 2021). We also admit the potential influence of the animal strains on the results of our study. Thus, C57BL/6N, DBA/2, and FVB/N mice were observed to vary significantly across sexes and anxiety, depression-like and social responses, and cognitive tasks under distinct behavioural paradigms (Knight et al. 2021; Pitzer et al. 2022). Specific experimental models may mirror numerous aspects and mechanisms of behaviour. There is a general concern to reproduce precisely psychiatric disorders in animals by virtue of complexity and involvement of higher nervous functions in humans. We, therefore, limited our study to a battery of the most commonly employed methods to evaluate the basic aspects of animal behaviour, however, a more expanded testing might shed a light on other consequences of DRD4 stimulation.

Altogether, the present study shows that the activation of DRD4 has little impact on anxiety-related and depression-like behaviour, nor on the reinforcing properties of alcohol in mice. However, the highly selective DRD4 ligand APH199 had an effect on psychotic-like behaviour in AMPH-sensitized rats by the aggravation of symptoms. APDs with DRD4 agonistic profile, developed to improve CIAS, may, thus, be potentially threatening because of the for effects on psychotic-like symptoms.

## Data Availability

Data are made available upon reasonable request.

## Abbreviations

ADHD: Attention deficit/hyperactivity disorder
AIH: Amphetamine-induced hyperlocomotion
AMPH: Amphetamine
ASR: Acoustic startle response
Bl: Baseline
CA: Closed arms
CC: Conditioning compartment
CPP: Conditioned place preference
DRD4: Dopamine receptors D4EPM—elevated plus maze
FST: Forced swim test
i.p.: Intraperitoneally
LDB: Light-dark box
NSF: Novelty suppressed feeding
OA: Open arms
OF: Open field
PC: Pseudoconditioning compartment
PPI: Prepulse inhibition
SD: Sprague–Dawley
SPT: Sucrose preference test
VEH: Vehicle
VNTR: Variable Number Tandem Repeats
